# Body Size Estimation from Early to Middle Childhood: Stability of Underestimation, BMI, and Gender Effects

**DOI:** 10.3389/fpsyg.2017.02038

**Published:** 2017-11-21

**Authors:** Silje Steinsbekk, Christian A. Klöckner, Alison Fildes, Pernille Kristoffersen, Stine L. Rognsås, Lars Wichstrøm

**Affiliations:** ^1^Department of Psychology, Norwegian University of Science and Technology, Trondheim, Norway; ^2^Institute of Psychological Sciences, University of Leeds, Leeds, United Kingdom; ^3^Human Development, NTNU Social Research, Trondheim, Norway

**Keywords:** children, body size estimation, underestimation, overweight, BMI, longitudinal design, bidirectional relationship, development studies

## Abstract

Individuals who are overweight are more likely to underestimate their body size than those who are normal weight, and overweight underestimators are less likely to engage in weight loss efforts. Underestimation of body size might represent a barrier to prevention and treatment of overweight; thus insight in how underestimation of body size develops and tracks through the childhood years is needed. The aim of the present study was therefore to examine stability in children’s underestimation of body size, exploring predictors of underestimation over time. The prospective path from underestimation to BMI was also tested. In a Norwegian cohort of 6 year olds, followed up at ages 8 and 10 (analysis sample: *n* = 793) body size estimation was captured by the Children’s Body Image Scale, height and weight were measured and BMI calculated. Overall, children were more likely to underestimate than overestimate their body size. Individual stability in underestimation was modest, but significant. Higher BMI predicted future underestimation, even when previous underestimation was adjusted for, but there was no evidence for the opposite direction of influence. Boys were more likely than girls to underestimate their body size at ages 8 and 10 (age 8: 38.0% vs. 24.1%; Age 10: 57.9% vs. 30.8%) and showed a steeper increase in underestimation with age compared to girls. In conclusion, the majority of 6, 8, and 10-year olds correctly estimate their body size (prevalence ranging from 40 to 70% depending on age and gender), although a substantial portion perceived themselves to be thinner than they actually were. Higher BMI forecasted future underestimation, but underestimation did not increase the risk for excessive weight gain in middle childhood.

## Introduction

Body image constitutes a perceptual and a subjective component; the former captures the accuracy of body size estimation, whereas the latter captures body satisfaction or dissatisfaction (Gardner, 1996; Wood et al., 1996; Gardner and Brown, 2010). The perceptual component is often referred to as overestimation or underestimation, indicating an inaccurate perception of body size (Cash and Pruzinsky, 2004; Hagman et al., 2015). Overestimation, which is related to anorexia nervosa (Gardner and Brown, 2014), has received far more attention in the literature compared to underestimation (Farrell et al., 2005), and the majority of studies on body size estimation have been conducted on adolescents and adults (McCabe et al., 2006; Sand et al., 2011; Cornelissen et al., 2015; Jackson et al., 2015; Liechty and Lee, 2015). However, because underestimation is related to being overweight (Maximova et al., 2008; Bodde et al., 2014), and more than one in three American children are overweight or obese (Singh et al., 2008; Ogden et al., 2014), with corresponding European numbers (Wijnhoven et al., 2014), underestimation in childhood needs to be examined. Obesity in children is a global health concern due to its effect on a range of disease related risk factors in childhood (Reilly, 2005; Russell-Mayhew et al., 2012), and because it strongly tracks into adulthood when negative effects on health are even more profound (Singh et al., 2008). Notably though, less than half of overweight youth consider themselves overweight or obese (Bodde et al., 2014). These numbers are concerning because overweight and obese underestimators are less likely to engage in weight loss efforts (Bodde et al., 2014). As suggested by theoretical models of health behavior change (Prochaska and Velicer, 1997) recognizing oneself as ‘at risk’ is necessary for behavior change to occur (Maximova et al., 2008). Thus, underestimation of body-size is a potential barrier to accomplish lifestyle changes needed to obtain a healthy weight. The association between higher weight and greater risk of body size underestimation (Saxton et al., 2009; Bodde et al., 2014) is also seen in children. A recent study found 86% of overweight and 62% of obese school aged children underestimated their body size, compared to 15% of normal weight children (Paul et al., 2015), which corresponds to earlier studies (Maximova et al., 2008). Further, the number of young people underestimating their body size is increasing: a recent study from 24 countries documented that rates of underestimation among overweight adolescents increased in 2009/2010 compared to 2001/2002 (Quick et al., 2014). The above-noted research is based on overweight samples, but to inform prevention of overweight we need insight into how underestimation of body size develops and tracks through childhood, and how weight-status relates to underestimation in community samples.

Cross-sectional research indicates that children are able to estimate the size of their body at an early age (Rolland et al., 1996; Gardner et al., 1997; Williamson and Delin, 2001; Truby and Paxton, 2002; Saxton et al., 2009) but prospective designs are needed to explore developmental trends at the group level and enable examination of individual stability/instability. To our knowledge, only two longitudinal studies of body size estimation in community samples of children exist (Gardner et al., 1999a,b; Allen et al., 2008). Allen et al. (2008) found over- and underestimation of body size at baseline to account for 12% of the variance in body size estimation 1 year later among 8 to 13 year olds (*n* = 259). Gardner et al. (1999a,b) measured body size estimation annually for 3 years beginning at ages 6, 9, and 12 (*n* = 189), and found a trend toward more underestimation with age which accords with cross-sectional research (Truby and Paxton, 2002). However, the number of children in each age group was small and predictors of stability in underestimation were not examined. The current inquiry extends the scant existing research by examining stability and change in children’s underestimation of body size, using a large and representative community sample of Norwegian 6 year olds, followed up at ages 8 and 10. Because underestimation is related to weight (Bodde et al., 2014), we examine BMI as a predictor of underestimation over time. Notably though, cross-sectional associations between BMI and underestimation could also indicate the opposite direction of influence, i.e., that underestimation predicts BMI. Surprisingly, a study of adolescents found underestimation of body size was linked to *reduced* risk of overweight or obesity 1 year later (Liechty and Lee, 2015), but it is not known if the same applies to children. The present study therefore also aims to examine the prospective paths from underestimation of body size to BMI.

Gender effects will also be explored. Teenage girls are more likely to be concerned about their weight than boys (Sweeting and West, 2002) and young girls have a greater desire to be thin compared to boys (Lowes and Tiggemann, 2003). They might also be more realistic, and thus accurate in their perception of body size. This is supported by studies showing boys are more likely to underestimate their weight status than girls (Brug et al., 2006; Zhao et al., 2012; Cattelino et al., 2015; Veldhuis et al., 2017). However, findings are inconsistent with one study reporting that girls underestimate their body size to a higher degree than boys (2002), while others found no gender differences (Gardner et al., 1997; Kornilaki, 2015).

In the present inquiry, the following hypothesis will be tested: (i) higher BMI will predict later underestimation, even when concurrent underestimation is taken into account; (ii) underestimation will not predict future BMI. We also aim to examine stability in underestimation from ages 6 to 10 years, but due to an insufficient evidence base, no hypothesis can be stated. The lack of consistent cross-sectional findings regarding gender differences also prevents clear predictions. Using a longitudinal design, we aim to explore whether prevalence of under- and overestimation differs between girls and boys from early to middle childhood, i.e., at ages 6, 8, and 10, and whether stability in underestimation differs by gender.

## Materials and Methods

### Participants and Procedure

**FIGURE 1:**
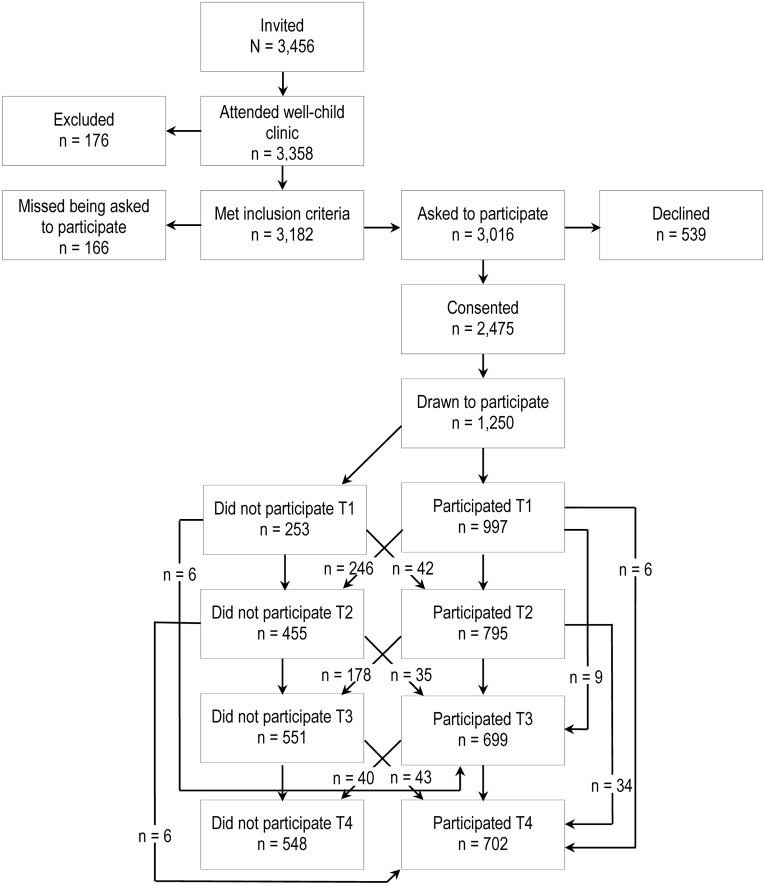
Study design.

A letter of invitation together with the Strengths and Difficulties Questionnaire (SDQ), a brief behavioral screening questionnaire, 4–16 version (Goodman et al., 2000) was sent to parents of two cohorts of children (born in 2003 or 2004) (*n* = 3456) living in Trondheim, Norway. Parents who consented to participate brought the completed SDQ to the ordinary community health checkup for 4-year-olds, where the health-care nurse informed the parents about the study using procedures approved by the Regional Committee for Medical and Health Research Ethics. Parents with insufficient proficiency in Norwegian to fill out the SDQ screening were excluded (*n* = 176). 82.1% of all the eligible parents agreed to participate and gave written informed consent (**Figure 1**). To increase variability and statistical power, children with high SDQ scores were oversampled, whereas those with low scores were undersampled. The SDQ was used because the primary aim of the Trondheim Early Secure Study (TESS), which the current inquiry is embedded in, was to assess mental health. We divided the SDQ scores into four strata (cut-offs: 0–4, 5–8, 9–11, and 12–40) and selected defined proportions of parents in each stratum to participate using a random-number generator. The probability of selection increased with increasing SDQ scores (0.37, 0.48, 0.70, and 0.89 in the four strata, respectively). As seen in **Figure 1**, based on this procedure 1250 participant were drawn to participate and 997 of these presented at the university clinic with their child for further testing (2007–2009) and thus constitute the sample at baseline. At follow-up 2 years later (T2) (2009–2011), 795 children participated (mean age = 6.7 years, *SD* = 0.17), whereas 699 children took part in the study at age 8 (T3) (2011–2013) (mean age = 8.8 years, *SD* = 0.24) and 702 at age 10 (T4) (2013–2015) (mean age = 10.51 years, *SD* = 0.17). The sample was comparable with the Norwegian parent population with regard to the parents’ level of education (Statistics Norway, 2012) and children’s BMI (Juliusson et al., 2013). Because body size estimations were included from T2 onward, the current inquiry uses data from T2 (6 years), T3 (8 years), and T4 (10 years) only. At T2, 50.3% of the participants were girls, mean BMI for the whole sample was 15.63 (95% CI = 15.43–15.69); 3.8% were categorized as overweight and 0.2% were obese according to the International Obesity Task Force cutoffs (IOTF) (International Obesity Task Force, 2011). Attrition analyses revealed that drop out was not predicted by gender, BMI, or estimation of body size.

### Measures

#### Body Size Estimation

Body size estimation was measured at all three time points using the Children’s Body Image Scale (CBIS), a figure rating scale validated for both genders (Truby and Paxton, 2002). The children were tested without the parents present. The measure was administrated by trained personnel with at least a bachelor’s degree in a relevant field and substantial practice in working with children and families. CBIS was developed for use in children aged 7 to 12, but has been successfully used with children as young as 5 years old (O’Connor et al., 2015). The scale, one for each gender, includes seven photographs of different body sizes. Every figure represents a defined BMI range, from picture one, the lowest BMI category (BMI = 14.0–14.6 for boys, 13.0–13.5 for girls) to picture seven, the highest BMI category (BMI = 28.5–29.0 for boys, 24.7–28.5 for girls). The child is asked to “select the photograph most similar to your own body size.” The discrepancy between actual body size and perceived body size is calculated by subtracting the number of the selected picture (e.g., 1) from the picture matching the child’s actual BMI (e.g., 7), thus the outcome have a possible range from -6 to +6. Based on this range, we categorized the scores from -2 to -6 as underestimation and from 2 to 6 as overestimation. Although the CBIS has good test–retest reliability (Truby and Paxton, 2008), a certain level of imprecision in children’s estimations is expected (Truby and Paxton, 2002). More specifically, if a child’s BMI falls close to the border of a pictorial category it would be of low relevance whether they chose the correct picture or the picture representing the bordering category. We therefore set the criteria for correct body size estimation to include one picture above or below the correct picture, i.e., -1 to 1.

#### Body Mass Index (BMI)

Digital scales were used to measure weight (Tanita BC420MA) and height (Heightronic digital stadiometer: QuickMedical, Model 235A) at all measurement points. Correction for light indoor clothing (0.5 kg for children) was applied. BMI was calculated (Cole et al., 1995). According to Cole et al. (2005), BMI is preferable in longitudinal designs, whereas BMI-z-score is optimal for cross-sectional assessments.

### Statistical Analysis

**FIGURE 2:**
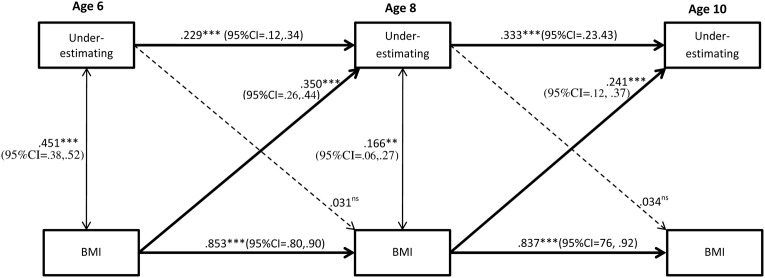
Stability of underestimation and BMI from ages 6 to 10 and the effect of BMI. Complete sample (*N* = 793). Significant paths are presented with straight lines, dotted lines represent non-significant paths. Ns = non-significant. All coefficients displayed are standardized. Between time paths = β; within time associations = *r*. ^∗∗^*p* < 0.01; ^∗∗∗^*p* < 0.001.

To examine predictors and stability of underestimation, as well as the paths from underestimation to BMI, logistic regression based cross-lagged analysis was applied with underestimation treated as a dummy variable (1 = underestimation, 0 = correct estimation). Because the aim of the study was to explore stability in *underestimation*, participants who correctly estimated, or overestimated their body size were excluded from this analysis. The latter was further due to the low prevalence of overestimation at ages 8 and 10 (see **Table 1**), which resulted in the study being underpowered to perform overestimation analyses with an acceptable degree of accuracy. In the cross-lagged model, underestimation and BMI at ages 8 and 10 were regressed on underestimation and BMI 2 years earlier, at ages 6 and 8 respectively (autoregressive paths). Underestimation at one time point was allowed to correlate with BMI at the same time point. To test if BMI was a predictor of underestimation over time, cross-lagged effects for BMI on underestimation were specified. To test the opposite direction of influence, paths from underestimation to BMI were specified. This allows us to analyze if changes in underestimation (and BMI respectively) can be explained by the status of BMI (and underestimation respectively) at an earlier point in time, over and above what is captured by stability in BMI and underestimation. All paths are displayed in **Figure 2**. To examine gender differences the analysis was repeated as a sub-group model for boys and girls. Wald tests were used to examine whether the detected paths differed by gender, testing one path at a time. A growth analysis was conducted to test if the hypothesized increase in underestimation from age 6 to 10 differed between boys and girls. The growth model yields two parameters; an intercept (underestimation at age 6) and a slope (growth in underestimation from age 6 to 10), which were both regressed on gender.

**Table 1:** Prevalence of boys and girls who underestimate, correctly estimate, or overestimate their body size at ages 6, 8, and 10.

Age	Gender	Underestimation	Correct estimation	Overestimation
6 years	Boys	20.6%	70.1%	9.3%
	Girls	20.0%	64.9%	15.0%
8 years	Boys	38.0%	59.5%	2.4%
	Girls	24.1%	73.6%	2.3%
10 years	Boys	57.9%	41.4%	0.7%
	Girls	30.8%	67.7%	1.4%


Analyses were performed in Mplus 7.4 (Muthèn and Muthèn, 1998–2010) using a robust maximum likelihood. Missing data were handled according to a full information maximum likelihood (FIML) procedure, which is considered to be state of the art and produces more correct estimates than complete case analysis (Schafer and Graham, 2002). FIML implies that analyses are performed on all available data, provided that cases have at least some values for the dependent variables (i.e., body size estimation at ages 6, 8, and 10). The analysis sample was therefore *n* = 793. Because we used a screen-stratified sample weighting according to a factor proportional to the number of children in the stratum divided by the number of children in that stratum was applied (i.e., undersampled children – those with low SDQ scores – were weighted up, whereas oversampled children – those with high SDQ scores – were weighted down) to provide accurate population estimates.

## Results

### Descriptive Analyses

**Table 1** displays the children’s body size estimation at ages 6, 8, and 10 years. Underestimation increased by age and was most prevalent in boys. Treating underestimation as a dummy variable (1 = underestimation, 0 = correct estimation), we found no significant gender difference with regard to the proportion of underestimators at age 6 (*z* = 0.30, *p* = 0.76). However, at ages 8 and 10, significantly more boys than girls underestimated their body size (Age 8: *z* = 4.33, *p* < 0.001, 0.01; Age 10: *z* = 7.60, *p* ≤ 0.001). Further, growth analyses revealed that girls had a less steep increase in underestimation from age 6 to 10 as compared to boys (*B* = -0.20, 95% CI = -0.36, -0.04; β = -0.52, *p* ≤ 0.01). To examine developmental differences in detail, the proportion of each gender who underestimated their body size at ages 6 and 8 were compared to the proportions of underestimators at ages 8 and 10, respectively. Significantly more boys underestimated their body size at age 8 compared to at age 6 (*z* = 4.93, *p* < 0.001). The proportion of male underestimators were also greater at age 10 compared to at age 8 (*z* = 5.40, *p* < 0.001), suggesting a clear trend toward more underestimating with increasing age. For girls, there was no difference in the proportion of underestimators at age 8 compared to at age 6 (*z* = 0.33, *p* = 0.74), but significantly more girls underestimated their body size at age 10 compared to at age 8 (*z* = 2.09, *p* = 0.037).

### Stability of Underestimation

**FIGURE 3:**
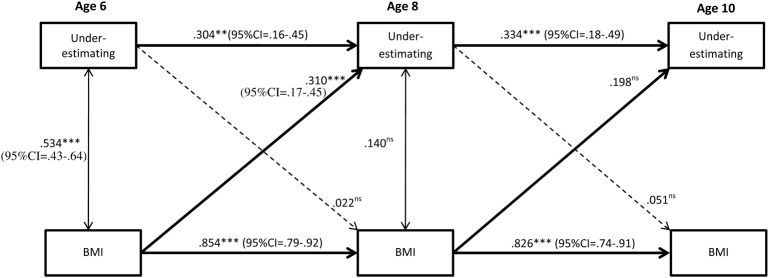
Stability of underestimation from ages 6 to 10 and the effect of BMI. Subgroup analysis of boys (*N* = 793). Significant paths are presented with straight lines, dotted lines represent non-significant paths. Ns = non-significant. All coefficients displayed are standardized. Between time paths = β; within time associations = *r*. ^∗∗^*p* < 0.01; ^∗∗∗^*p* < 0.001.

**FIGURE 4:**
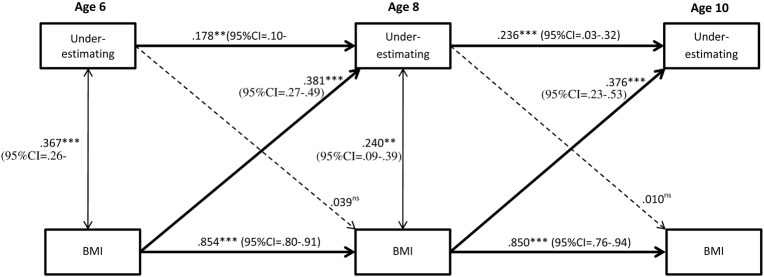
Stability of underestimation from ages 6 to 10 and the effect of BMI. Subgroup analysis of girls (*N* = 793). Significant paths are presented with straight lines, dotted lines represent non-significant paths. Ns = non-significant. All coefficients displayed are standardized. Between time paths = β; within time associations = *r*. ^∗∗^*p* < 0.01; ^∗∗∗^*p* < 0.001.

As can be seen in **Figure 2**, there was stability of underestimation, albeit low, from age 6 to age 8, and from 8 to 10. Wald test of parameter constraints revealed that the stability from age 6 to 8 was significantly lower compared to the stability from age 8 to 10 (Wald = 27.82, df = 1, *p* ≤ 0.001), suggesting an increasing stability in underestimation by age. Stability in underestimation was evident for both genders and did not significantly differ between boys and girls (age 6 to age 8: Wald = 1.67, df = 1, *p* = 0.20; Age 8 to age 10: Wald = 0.44, df = 1, *p* = 0.51). **Figures 3**, **4** display the results of the gender specific analysis for girls and boys respectively.

### BMI as a Predictor of Stability in Underestimation

As shown in **Figure 2**, higher BMI predicted underestimation. More specifically, higher BMI at age 6 increased the odds for being an underestimator at age 8, and higher BMI at age 8 predicted underestimation at age 10, even when stability of underestimation was taken into account. The subgroup (i.e., gender-specific) analyses revealed that age 6 BMI predicted underestimation at age 8 for both genders, but the prediction from age 8 BMI to underestimation at age 10 was evident in girls only (**Figures 3**, **4**). However, the Wald test of parameter constraints revealed no significant gender difference with regard to BMI as a predictor of underestimation (Age 6 BMI to underestimation age 8: Wald = 0.18, df = 1, *p* = 0.67; Age 6 BMI to underestimation age 8: Wald = 1.28 df = 1, *p* = 0.26). The overall model and the subgroup analyses for the reverse direction of association revealed that underestimation did not predict future BMI.

## Discussion

This prospective community study revealed that more children underestimated than overestimated their body size at ages 6, 8 and 10 years, and overestimation virtually disappeared from age 8 onward. Underestimation was modestly stable and was predicted by higher BMI. Significantly more boys than girls underestimated their body size at ages 8 and 10.

### Low Individual Stability in Underestimation of Body Size

Individual stability in underestimation was significant, but modest from ages 6 to 8 and 8 to 10, although the stability significantly increased with age. Our findings accord with those of Gardner et al., 1999a, who reported low test–retest consistency of body size estimation using a video-adjustment technique in a sample of children aged 6–12, followed up annually for 3 years. The rate of over and underestimation at age 6 and the low test–retest concur with a view that body size estimation is not yet firmly established during the first school years. Hence, reliability is expected to be low and it is therefore not surprising that stability is modest, especially over longer periods, i.e., in our case 2 years. Increasing awareness with age, and thus increasing reliability, however, should be reflected in increasing stability; also concurring with our results.

### BMI Predicts Underestimation of Body Size

Our study adds to earlier cross-sectional research (Maximova et al., 2008; Bodde et al., 2014) and one prospective study with a 1-year follow-up (Allen et al., 2008) by showing that higher BMI predicts future underestimation. Underestimation could be seen as an adaptive mechanism. Not acknowledging your own overweight may help protect against psychosocial consequences of obesity (Armstrong et al., 2014). In support of this view, research shows that overweight or obese youth who accurately perceive their weight are more likely to be depressed (Gray et al., 2012) and report more psychological distress (Xie et al., 2011) compared to those who not see themselves as overweight.

Our study is the first to demonstrate that underestimation does not constitute a risk factor for weight gain in middle childhood, as has previously been shown in adolescents and adults (Liechty and Lee, 2015; Robinson et al., 2015). Overall, our findings extend earlier cross-sectional research by suggesting that the association between underestimation and weight status is due to the effect of weight on underestimation, not the other way around. Thus, although underestimation of body-size is considered a potential barrier to obtain a healthy weight, our findings indicate that it is not so in normally developing children. A potential implication of these findings is that there may be no reason to promote a more correct perception of body size in healthy children who underestimate, at least not in order to prevent future excessive weight gain. However, it is not known whether this also applies to children with overweight or obesity.

### Gender Differences in Body Size Estimation

At ages 6 and 8, about two thirds of both boys and girls correctly estimated their body size; comparable proportions to those found in earlier research (Gardner et al., 1999b; Saxton et al., 2009). However, at 10 years of age only 41% of boys accurately estimated their body size, compared with 68% of girls. Others also report boys to be less accurate than girls (2008), although some studies report no gender difference in the accuracy of body size estimation (Gardner et al., 1997; Kornilaki, 2015). We found more boys than girls underestimated their body size at ages 8 and 10. In a sample consisting of 199 children under the age of 10, Truby and Paxton (2002) report boys to be *less* likely to underestimate their body size compared to girls, but studies of weight perception applying larger samples do report that boys are more likely to underestimate than girls (Zhao et al., 2012). Nevertheless, further research is needed before firm conclusions can be drawn regarding gender differences in body size estimation.

Although more boys than girls were found to underestimate as they age, the individual stability in underestimation did not differ between the genders. Moreover, there was no gender difference in the effect of BMI on underestimation. These results suggest the mechanisms of underestimation are not gender specific.

### Limitations

The longitudinal design, the large, population-representative sample, and objectively measured height and weight are strengths of the current study. Notably however, several individual and environmental factors not examined in our study may also affect children’s underestimation of body size. For example, research shows greater schoolmate BMI to be significantly associated with underestimation of weight status among children and adolescent (Maximova et al., 2008), and common genetics cannot be ruled out. Further, because the present sample consists of Caucasian children, as do earlier studies applying CBIS (Truby and Paxton, 2008), caution should be applied in generalizing the findings to other ethnic groups and cultures for whom body ideals may vary (Gordon et al., 2010). Future studies should aim to replicate the findings in divergent samples of school aged children, also among overweight and obese.

## Summary and Conclusion

The majority of 6, 8, and 10-year old children correctly estimate their body size. Even so, a substantial portion, particularly among boys and at older ages, perceived themselves to be thinner than they actually were, whereas hardly any overestimated their body size from age 8 onward. At age 10, boys were more likely to underestimate than accurately estimate their body size. Stability of underestimation was low, especially at younger ages. Higher BMI predicted future underestimation, but not the other way around.

## Ethics Statement

This study was carried out in accordance with the recommendations of the Regional Committee for Medical and Health Research Ethics – Central Norway, with written informed consent from all subjects. All subjects gave written informed consent in accordance with the Declaration of Helsinki. The protocol was approved by the Regional Committee for Medical and Health Research Ethics – Central Norway.

## Author Contributions

SS and LW were responsible for the study concept and design. SS carried out initial analysis and drafted the manuscript based on input from PK, SLR and AF. SS, LW, and CK conducted the statistical analysis, whereas LW obtained funding. All authors contributed to the interpretation of data, critically revised the manuscript for important intellectual content, approved the final manuscript as submitted and agreed to be accountable for all aspects of the work.

## Conflict of Interest Statement

The authors declare that the research was conducted in the absence of any commercial or financial relationships that could be construed as a potential conflict of interest.
